# Long Noncoding RNA SNHG16 Regulates the Growth of Human Lung Cancer Cells by Modulating the Expression of Aldehyde Dehydrogenase 2 (ALDH2)

**DOI:** 10.1155/2022/2411642

**Published:** 2022-05-20

**Authors:** Yan Li, Lifeng Jiang, Zhaocheng Zhu, Binfan Fu, Xu Sun, Yue Jiao

**Affiliations:** Department of Integrated Traditional Chinese-Western Medicine, The Affiliated Cancer Hospital of Zhengzhou University, Henan Cancer Hospital, Zhengzhou, Henan, China 450008

## Abstract

The involvement of long noncoding RNA (lncRNA) SNHG16 has been reported in several human cancers. Notwithstanding, the role of lncRNA SNHG16 is yet largely unknown in human lung cancer. Consequently, this study was undertaken to investigate the role and therapeutic potential of SNHG16 in human lung cancer. The results showed a significant (*P* < 0.05) transcriptional upregulation of SNHG16 in lung cancer tissues and cell lines. However, downregulation of SNHG16 resulted in significant (*P* < 0.05) inhibition of lung cancer A549 and SK-LU-1 cell proliferation. DAPI and annexin V/PI assays revealed apoptosis to be responsible for inhibition of cell proliferation and colony formation observed upon SNHG16 knockdown. This was accompanied by enhancement of Bax and suppression of Bcl-2 expression in A549 and SK-LU-1 cells. Transwell assays revealed that silencing of SNHG16 also significantly (*P* < 0.05) inhibited migration and invasion of A549 and SK-LU-1 cells. Bioinformatic analysis revealed that SNHG16 interacted with ALDH2 to exert its effects in human lung cancer cells. The expression of ALDH2 was found to be significantly (*P* < 0.05) suppressed in human lung cancer tissues and cell lines. Overexpression of ALDH2 inhibited the proliferation and colony formation of the A549 and SK-LU-1 cells. However, silencing of ALDH2 could avoid the tumor-suppressive effects of SNHG16 knockdown. Finally, SNHG16 silencing was also found to inhibit in vivo tumor growth. Collectively, the study unveils the molecular role of SNHG16 in regulating the development of lung cancer by interacting with ALDH2.

## 1. Introduction

Lung cancer is one of prevalent and most fatal human cancers responsible for the highest number of cancer-related deaths at the global level [1]. In 2020 alone, about 0.116 million men and 0.112 million women were affected by lung cancer, and more than 0.13 million patients died from the underlying malignancy in the United States [2]. At present, approximately 8.2 million deaths are believed to result from lung cancer annually, and the figures are alarmingly suggested to approach the 10 million per annum mark by 2030 [3]. The figures thus suggest the inefficiency of the presently opted anticancer measures against the malignancy of lung cancer and emphasize the necessity of exploring the alternative treatment measures which could help in restricting the lethality of lung cancer more effectively. The advancement in the sequencing techniques and microarray analysis along with the availability of diversity of bioinformatics software tools has been shown that more than 90% of human genome does not code for proteins although it is actively transcribed into RNA species called noncoding RNAs [4, 5]. Falling in the latter category, the long noncoding RNAs (lncRNAs) are greater than 200 nucleotides in length and help in gene regulation at transcriptional or posttranscriptional level depending on subcellular localization of lncRNAs [6]. Interestingly, the lncRNA dysregulation has been shown to be linked to a number of human cancers [7–9]. Also, a vast number of lncRNAs have been found to be differentially expressed in lung cancer tissues when compared to normal lung tissue samples implying their probable involvement in lung cancer growth and development [10–12]. The recently identified lncRNA, small nucleolar RNA host gene 16 (SNHG16), has been found to be involved in the progression of number of human cancers like colon and cervical cancers [13, 14]. LncRNA SNHG16 has also been found to be involved in the development of lung cancer. For instance, increased expression of SNHG16 has been shown to be associated with poor prognosis of lung cancer [15]. Guo et al. reported that SNHG16 promoted the proliferation and invasion of lung adenocarcinoma cells via sponging of let-7a-5p [16]. Similarly, Yu et al. showed the involvement of SNHG16 in the development of non-small-cell lung cancer development [17]. In other studies, SNHG16 has been shown to promote proliferation of lung cancer via miR-520/VEGF axis [18] and USP21/YY1 axis [19]. However, the role of lncRNA SNHG16 in lung cancer via modulation of ALDH2 expression has not been studied and was thus the objective of the present study.

## 2. Materials and Methods

### 2.1. Tissue Specimen and Cell Lines

Clinical specimens comprising of normal and malignant lung tissues were obtained from a total of 30 patients after surgical resection without chemo- or radiotherapy application at Henan Cancer Hospital. The tissues were collected from the patients after informed consent. This study was approved by the Ethical Committee of Henan Cancer Hospital under approval number HCH-63/2019. The pathological staging of the lung cancer tissues was performed by the hospital pathologists. The characteristics of the patients are listed in Table 1. All the clinical specimens were stored at -80°C until experimental use. Four lung cancer cell lines (A427, A549, HCC827, and SK-LU-1) and a normal lung cell line (MRC5) were obtained from the American Type Culture Collection. All the cell lines were cultured using DMEM (Gibco) medium at 37°C with 5% CO_2_ concentration. Humidified incubator was used for maintenance of the cell lines.

### 2.2. Transfection

The transfection of cell lines was carried out through Lipofectamine 2000 (Thermo Fisher Scientific)–based method following the manufacturer's guidelines. The small interfering RNA (siRNA) constructs of SNHG16 (si-SNHG16) and ALDH2 (si-ALDH2) along with its negative control (si-NC) were purchased from RiboBio Co., Ltd (Guangzhou, China). Mammalian overexpression vector, pcDNA3.1, was used for generating the overexpression construct of ALDH2.

### 2.3. Expression Analysis

To extract total RNA from tissues and cell lines, the samples were processed through TRIzol reagent (Thermo Fisher Scientific)–based method as per the manufacturer protocol. The RNA was reverse transcribed to synthesize first-strand cDNA with the help of PrimeScript 1st strand cDNA synthesis kit (Takara Bio). The SYBR Green PCR mix (Thermo Fisher Scientific) was used to perform real-time PCR (RT-PCR) on QuantStudio 5.0TM RT-PCR system (Thermo Fisher Scientific) to analyze the relative gene expression levels of SNHG16 and ALDH2. The 2^-ddCt^ method was used for determining the relative expression values. Actin and GADPH genes were used as an internal expression control in RT-PCR study. The nucleotide sequences of the primers used in the study are listed in Table 2.

### 2.4. Cell Viability Assay

The transfected A549 and SK-LU-1 cancer cells were added to 96-well microtiter plates with cellular density of 5,000 cells/well. The cells were cultured for 0, 12, 24, 48, and 96 h at 37°C. Afterwards, the wells were inoculated with MTT reagent (Thermo Fisher Scientific) at final concentration of 5 mg/mL culture medium, and incubation at 37°C was prolonged for 4 h. Then, 250 *μ*L of DMSO were added to each well for dissolving the formazan precipitate, and finally the absorbance was recorded per sample at 570 nm. Three replicas were kept for each experimental sample.

### 2.5. Colony Formation Assay

The colony forming ability of A549 and SK-LU-1 cells after transfection with suitable constructs was determined by plating 500 cells/well (in a 6-well dish) in their regular media for about 12 days. Thereafter, the cells were stained with 0.5% crystal violet (in methanol) for 5 minutes. Following staining, the cells were washed in water, and the plates were dried overnight. Colonies with more than 50 cells were counted as a clone.

### 2.6. Cell Apoptosis Assays

The cancer cells were propagated in 96-well plates at 2 × 10^5^ cells/well. Afterwards, the cell clusters were transfected with suitable constructs and then incubated for 24 h at 37°C. The cells were then harvested, fixed in 70% ethanol for 25 min at room temperature, washed three times with PBS, and subsequently treated with 0.3% Triton X-100 for 10 min for permeabilization. DAPI solution was used to stain the cells. The stained cells were dark incubated for 15 min and PBS washed. Finally, the DAPI signals were investigated through Cytation 5 Cell Imaging Multi-Mode Reader (BioTek, VT).

The apoptosis of transfected cancer cells was determined using Annexin V-FITC/PI apoptosis detection kit (BD Biosciences) as per manufacturer protocol. In brief, binding buffer was used for resuspending the transfected cells which were then stained with 15 *μ*L annexin V-fluorescein isothiocyanate (FITC) and propidium iodide (PI). Finally, flow cytometry was performed to determine the percentage of apoptotic cells.

### 2.7. Transwell Assay

A 24-well Transwell plate fitted without or with Matrigel, respectively, was used to analyze the migration and invasion of the transfected A549 and SK-LU-1 lung cancer cells transfected with si-NC or si-SNHG16. The cells were placed in the upper chamber of the Transwell plate, and the lower chamber was added with DMEM growth medium only. Following 24-h incubation at 37°C, the cells invading the lower chamber were fixed with methanol and then stained with 0.1% crystal violet. Lastly, the cells were examined under the light microscope (100×), photographs were taken, and randomly selected microscopic fields were used to determine the percentage of migration and invasion of cancer cells.

### 2.8. Wound Healing Assay

Transfected A549 and SK-LU-1 cells were cultured to obtain 90% confluency. A wound was created by scraping the cells using a P200 pipette tip, and images were captured immediately (0 h) and after 24 h. Migration of cells was assessed by observing the width of the wounds.

### 2.9. In Silico Target Analyses and Dual Luciferase Assay

In silico analyses were performed using LncRRIsearch software (http://rtools.cbrc.jp/LncRRIsearch/detail.cgi) and VARNA: Visualization Applet for RNA software (http://varna.lri.fr/) to predict the interaction between SNHG16 and ALDH16. In silico analyses were performed using LncRRIsearch software (http://rtools.cbrc.jp/LncRRIsearch/detail.cgi) and VARNA: Visualization Applet for RNA software (http://varna.lri.fr/) to predict the interaction between SNHG16 and ALDH16. The dual luciferase reporter assay was performed to confirm the in silico results. The A549 lung cancer cells were cotransfected with overexpression construct of SNHG16 (pcDNA-SNHG16) or control vector (pcDNA3.1) and luciferase reporter construct carrying 3′-UTR fragment of ALDH2 either in native (WT) or mutated state (MUT). The luciferase activity was measured using Dual-Luciferase Reporter Assay System (Promega) using Renilla luciferase gene as control of luciferase activity.

### 2.10. Western Blotting

The A549 and SK-LU-1 cancer cells stably transfected with si-SNHG16 or si-NC were lysed using the RIPA lysis and extraction buffer (Thermo Fisher Scientific) to extract total cellular proteins. After total protein concentration estimation, equal protein counts were separated using SDS-PAGE and then blotted to PVDF membranes. The protein bands were visualized through chemiluminescence following the treatment with specific primary and secondary antibodies. The western blotting study was normalized using the human *β*-actin protein.

### 2.11. In Vivo Study

Around 5-week-old BALB/c nude mice were used to create xenograft tumors in vivo by transplanting A549 lung cancer cells. 10^3^ to 10^4^ A549 lung cancer cells were suspended in Matrigel (BD Biosciences, USA) and then injected into the right flank mice, subcutaneously. After 2 weeks of the cell implantation, the mice were intraperitoneally administered with si-SNHG16 or negative control. Tumor volume was monitored every third day continuously for 3 weeks. After 3 weeks, the mice were euthanized, and the tumor weight was estimated. The 10% paraformaldehyde was used for fixing the tumors to be used for further investigation. The experimental use of animals was approved by the Institutional Ethics Committee. Standard institutional guidelines and scientific regulations were strictly followed for the experimental usage of animals.

### 2.12. Hematoxylin and Eosin (H&E) Staining and Immunohistochemical Staining

Formalin-fixed tumor tissues from nude mice were embedded in paraffin and cut into thin sections. The tissue sections were subsequently subjected to staining with H&E using standard procedures. For immunohistochemical staining, tissue sections were dewaxed and subsequently rehydrated in graded ethanol. Microwave heating in sodium citrate buffer (pH 6.0) was carried out for antigen retrieval. The sections were immersed in 3% H_2_O_2_ for 10 min to block endogenous peroxidase activity and subsequently subjected to incubation with primary antibodies at 4°C overnight. After washing, the sections were incubated with HRP-conjugated secondary antibody at room temperature for 1 h, and reactive products were visualized by staining with 3,3′-diaminobenzidene (DAB). The images were obtained under a microscope (Olympus, Japan) with appropriate magnification.

### 2.13. Statistical Analysis

The final values represent the mean ± standard deviation (SD) determined using three replicas per experimental setup. One-way ANNOVA or Student's *t*-test was performed using GraphPad Prism 7.0 software for statistical analysis. The *P* value at <0.05 was taken as the measure of statistically significant difference.

## 3. Results

### 3.1. Transcriptional Upregulation of SNHG16 in Lung Cancer

**Figure 1:**
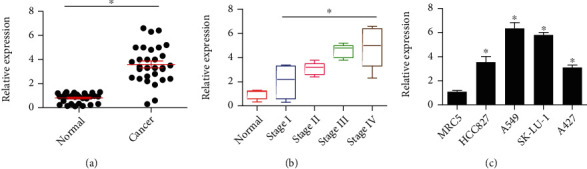
LncRNA-SNGH2 has significant upregulation in lung cancer. Expression of SNGH2 in (a) normal and cancerous lung tissues, (b) clinical tissues comprising of different pathological stages of lung cancer, and (c) normal lung cell line (MRC5) and lung cancer cell lines (A427, A549, HCC827, and SK-LU-1) as determined by qRT-PCR. The gene expression study was performed using three replicates (∗*P* < 0.05).

The results revealed the expression of SNH16 gene expression was significantly (*P* < 0.05) higher in lung cancer tissues in comparison to the normal lung tissues (Figure 1(a)). Further, SNHG16 expression was found to be enhancing with the progression of each pathological stage of lung cancer (Figure 1(b)). The lung cancer cell lines (A427, A549, HCC827, and SK-LU-1) were also found to exhibit significantly (*P* < 0.05) higher SNHG16 expression relative to the normal lung cell line (MRC5) (Figure 1(c)). Next the clinical relevance of SNH16 expression and clinicopathological features of the patients were studied. For this, the samples were divided into two groups based on their average expression of SNHG16; a high expression group, and a low expression group. It was found that SNHG16 expression was positively associated with lymph node metastasis, distant metastasis, and clinical stage (Table 1). Collectively, these findings indicated a possible regulatory role of SNHG16 in lung cancer.

### 3.2. Decline in Cell Viability under SNHG16 Downregulation

**Figure 2:**
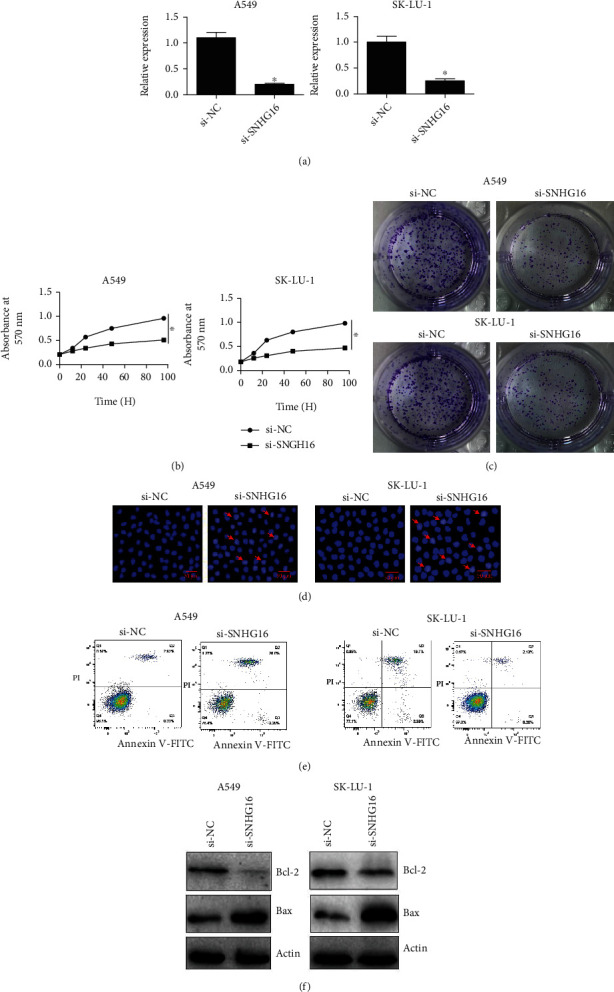
SNHG16 repression reduced the viability of lung cancer cells through apoptosis. (a) Confirmation of SNHG16 transcriptional knockdown in A549 and SK-LU-1 lung cancer cells, (b) determination of viabilities of A549 and SK-LU-1 lung cancer cells transfected with si-NC or si-SNHG16 by MTT assay, (c) colony formation assay showing colony formation of A549 and SK-LU-1 cells transfected with si-NC or si-SNHG16, (d) DAPI staining of A549 and SK-LU-1 lung cancer cells transected with si-SNHG16 or si-NC (arrows depict apoptotic cells), (e) flow cytometric analysis for detection of apoptosis of A549 and SK-LU-1 lung cancer cells transected with si-SNHG16 or si-NC, and (f) western blotting of Bax and Bcl-2 marker proteins form A549 and SK-LU-1 lung cancer cells transected with si-SNHG16 or si-NC. Each experiment was performed in triplicates, and difference between two values was considered significant at ∗*P* < 0.05.

To understand the growth regulatory role of SNHG16 in lung cancer, lung cancer cell lines A549 and SK-LU-1 were transfected with si-SNHG16, and downregulation of SNHG16 was confirmed from the transfected cancer cells in comparison to the negative control cells (Figure 2(a)). The cancer cells were then processed for viability assessment using MTT assay, and it was shown that cancer cells exhibited significant (*P* < 0.05) decline in their viabilities under SNHG16 knockdown (Figure 2(b)). Knockdown of SNHG16 also caused considerable decrease in the colony formation potential of A549 and SK-LU-1 cells (Figure 2(c)). Next, A549 and SK-LU-1 lung cancer cells transfected with si-SNHG-16 along with the respective negative control cancer cells were analyzed for nuclear morphology using DAPI staining. Both the cell lines exhibited apoptotic signs and showed loss of nuclear integrity (Figure 2(d)). To further confirm the induction of apoptosis in cancer cells under SNHG16 silencing, flow cytometric analysis was used. The percentage of apoptotic cells was comparatively higher under SNHG16 silencing (Figure 2(e)). From the western blotting, it was found that Bax protein concentration was considerably higher, while reverse was true for Bcl-2 under SNHG16 knockdown (Figure 2(f)). Together, the results are indicative of apoptosis mediated loss of lung cancer cell viability under SNHG16 gene silencing.

### 3.3. SNHG16 Silencing Inhibits Cell Migration and Invasion

**Figure 3:**
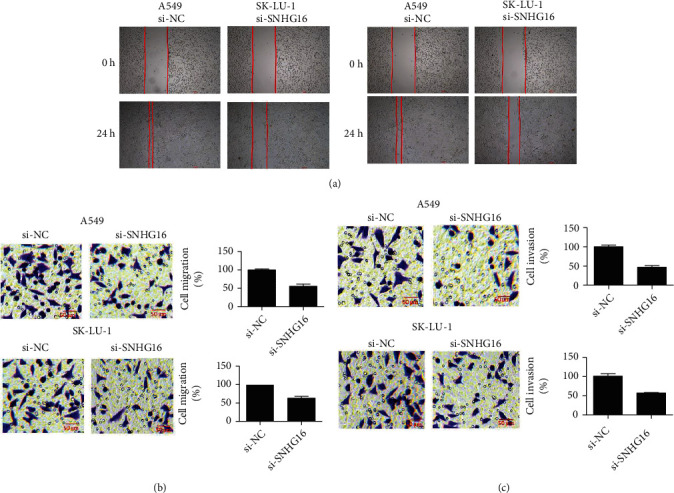
SNHG16 repression inhibits the migration and invasion of lung cancer cells. (a) Wound healing assay of si-NC or si-SNHG16 transfected A549 and SK-LU-1 cells. (b) Transwell assay showing the cell migration of si-NC and si-SNHG16 transfected A549 and SK-LU-1 cells. (c) Transwell assay showing the cell invasion of si-NC and si-SNHG16 transfected A549 and SK-LU-1 cells. Each experiment was performed in triplicates (∗*P* < 0.05).

The migration and invasion of the lung cancer cell lines, A549, and SK-LU-1 transfected with si-NC or si-SNHG16 were estimated through wound healing and Transwell chamber assays. The results of wound healing assay showed that knockdown of SNHG16 prevented the migration of A549 and SK-LU-1 cells (Figure 3(a)). These results were also supported by the results of Transwell assay which showed that silencing of SNHG16 decreased the migration of A549 and SKLU-1 cells by around 45% (Figure 3(b)). Similarly, the invasion of the A549 and SKLU-1 cells was inhibited by around 50% (Figure 3(c)).

### 3.4. Interaction of SNHG16 with ALDH2 in Lung Cancer

**Figure 4:**
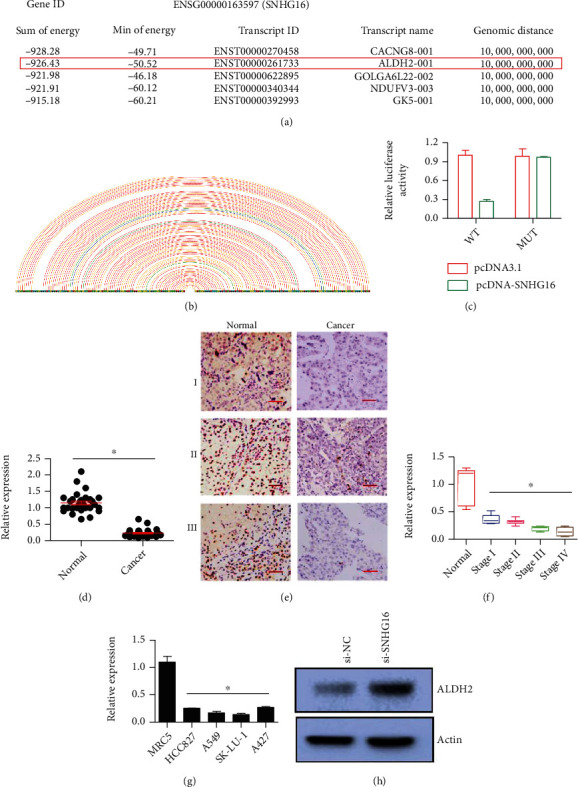
SNHG16 interacted and repressed ALDH2 in lung cancer, posttranscriptionally. (a) In silico analysis for prediction of SNHG16 and ALDH2 interaction in lung cancer through LncRRIsearch software; (b) in silico analysis for prediction of SNHG16 and ALDH2 interaction in lung cancer through VARNA: Visualization Applet for RNA software; (c) dual luciferase assay showing interaction between SNHG16 and ALDH2; (d) relative gene expression of ALDH2 in normal and cancerous lung tissues; (e) IHC analysis showing expression of ALDH2 in three pairs (I, II, and III) of normal and cancerous tissues; (f) relative gene expression of ALDH2 at different pathological stages of lung cancer; (g) relative gene expression of ALDH2 in normal lung cell line (MRC5) and lung cancer cell lines (A427, A549, HCC827, and SK-LU-1); (h) relative protein expression of ALDH2 in A549 cancer cells transfected with si-SNHG16 or si-NC. Each experiment was performed with three replicates (∗*P* < 0.05).

The interaction of SNHG16 with ALDH2 was predicted through in silico analyses (Figures 4(a) and 4(b)). The in silico results were confirmed through dual-luciferase reporter assay where the interaction of SNHG16 with UTR of ALDH16 was confirmed from the decline of luciferase activity (Figure 4(c)). For further confirmation, we evaluated the expression of ALDH2 in lung cancer tissues and cell lines by qRT-PCR. It was found that the expression pattern of ALDH2 in lung cancer tissues was also found to be downregulated in human ALDH2 in lung cancer cell lines (Figure 4(d)). IHC analysis also showed considerable downregulation of ALDH2 in lung cancer tissues (Figure 4(e)). Moreover, the expression of ALDH2 decreased with advancement of the disease (Figure 4(f)). The expression of ALDH2 was also downregulated in human lung cancer cell lines (Figure 4(g)). However, overexpression of SNHG16 in A549 lung cancer cells increased the protein concentration of ALDH2 in lung cancer cells (Figure 4(h)). Additionally, SNH16 was found to be negatively correlated (*r* = −0.812) with ALDH2 expression, and the upregulation of SNH16 was manifested as transcriptional repression of ALDH2 suggesting the modulation of molecular role of SNH16 through ALDH2 targeting in lung cancer.

### 3.5. Exertion of SNHG16 Role through ALDH2 in Lung Cancer

**Figure 5:**
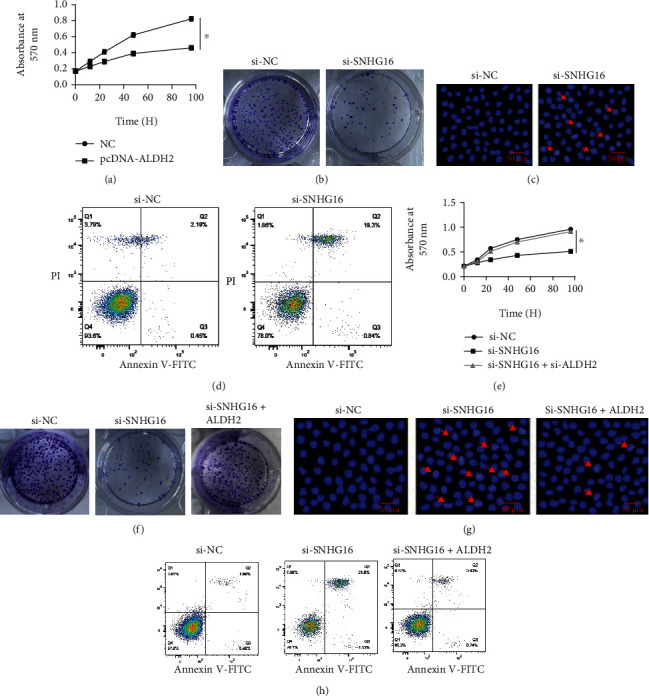
ALDH2 modulated the regulatory role of lncRNA-SNHG16 in lung cancer. (a) Estimation of viabilities of A549 cancer cells by MTT assay at different culture stages, transfected with pcDNA-ALDH2 or control vector pcDNA3.1 (NC); (b) colony formation of A549 cancer cells transfected with pcDNA-ALDH2 or control vector pcDNA3.1 (NC); (c) DAPI staining of A549 cancer cells transfected with pcDNA-ALDH2 or NC; (d) flow cytometry for analysis of apoptosis of A549 cancer cells transfected with pcDNA-ALDH2 or NC; (e) estimation of viabilities of A549 cancer cells by MTT assay at different culture stages, transfected with si-SNHG16, pcDNA-ALDH2, or si-SNHG16 plus si-ALDH2; (f) colony formation of A549 cancer cells transfected with si-SNHG16, pcDNA-ALDH2, or si-SNHG16 plus si-ALDH2; (g) DAPI staining of A549 cancer cells transfected with si-SNHG16, si-NC, or si-SNHG16 plus si-ALDH2; (h) flow cytometry for analysis of apoptosis of A549 cancer cells transfected with si-SNHG16, si-NC, or si-SNHG16 plus si-ALDH2. Three replicates were used for performing the experiments (∗*P* < 0.05) (arrows depict apoptotic cells).

To confirm whether SNHG16 exerted its growth regulatory role in lung cancer through negative regulation of ALDH2, the overexpression of ALDH2 was performed in lung cancer cells. The lung cancer cell viabilities and colony formation was found to be significantly (*P* < 0.05) lower under ALDH2 overexpression (Figures 5(a) and 5(b)). Cancer cells overexpressing ALDH2 were inducted for apoptosis (Figures 5(c) and 5(d)). Further, inhibition of ALDH2 could avoid the inhibitory effects of SNHG16 knockdown on viability and colony formation of A549 cells (Figures 5(e) and 5(f)). Consistently, the lung cancer cells did not show apoptotic features when cotransfected with silencing constructs of ALDH2 and SNHG16 (Figures 5(g) and 5(h)). The results thus confirmed the modulation of growth regulatory effects of SNHG16 in lung cancer through ALDH2 posttranscriptional downregulation.

### 3.6. Rescue Effects of ALDH2 Silencing on Migration and Invasion of Lung Cancer Cells under SNHG16 Knockdown

**Figure 6:**
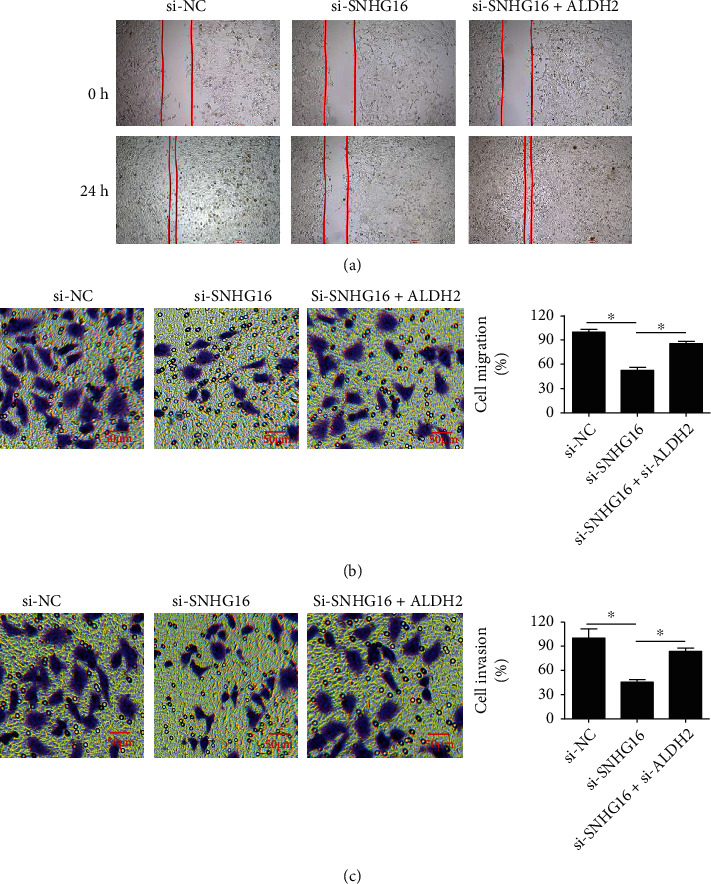
Rescue effect of ALDH silencing on invasion of lung cancer cells under SNHG16 knockdown: (a) wound healing assay showing migration in A549 cells transfected with si-NC, si-SNHG16, and si-SNHG16 + si-ALDH; (b) Transwell assay showing cell migration of A549 cells transfected with si-NC, si-SNHG16, and si-SNHG16 + si-ALDH; (c) Transwell cell invasion of A549 cells transfected with si-NC, si-SNHG16, and si-SNHG16 + si-ALDH. Three replicates were used for performing the experiments (∗*P* < 0.05).

The effects of ALDH2 silencing were evaluated on the migration and invasion of A549 cells under SNHG16 knockdown by wound healing and Transwell assays. It was found that migration and invasion of A549 cells was significantly (*P* < 0.05) inhibited under SNHG16 knockdown. However, silencing of ALDH2 could prevent the inhibitory effects of SNHG16 on the migration and invasion of A549 cells (Figures 6(a)–6(c)).

### 3.7. Silencing of SNJG16 Restrained In Vivo Tumor Growth and Upregulated ALDH2

**Figure 7:**
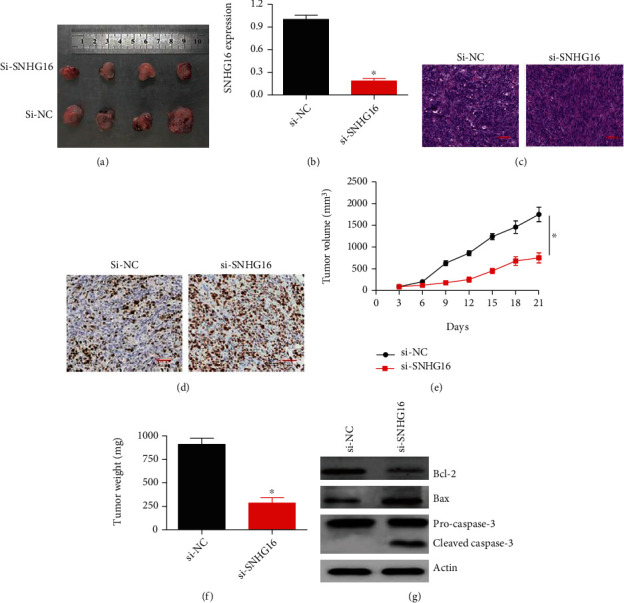
SNHG16 knockdown suppresses in vivo mice tumor xenograft growth. (a) Analysis of tumor size from the mice administered with si-SNHG16 or si-NC; (b) expression of SNHG16 in mice tumors administered with si-SNHG16 or si-NC; (c) H&E staining of si-NC and si-SNHG16 tumors; (d) IHC showing expression of ALDH2 protein in si-SNHG16 or si-NC tumors; (e) analysis of tumor volume at indicated study stages from the mice administered with si-SNHG16 or si-NC; (f) average weight of si-SNHG16 or si-NC tumors; (g) western blotting showing expression of Bax, Bcl-2, and cleaved caspase-3 in si-NC or si-SNHG16 tumors (∗*P* < 0.05).

The investigation of tumor regulatory role SNHG16 was carried out using the mice xenograft models. The mice tumor xenografts were constituted by injecting the animals with A549 cancer cell suspension, subcutaneously. Subsequently, the intraperitoneal (IP) injections were given carrying si-SNHG16 or its negative control, si-NC. The administration of mice with si-SNGH16 inhibited the xenograft tumor growth, significantly (Figure 7(a)). The SNGH16 silencing was reconfirmed by qRT-PCR (Figure 7(b)). H&E staining showed smaller and more unified nuclei in the si-SNHG16 tumors (Figure 7(c)).

IHC staining showed mice tumors injected with si-SNHG16 constructs exhibited considerable upregulation of ALDH2 (Figure 7(d)). The mice tumor volume was monitored every 3rd day after cancer cell implantation. The average mice tumor volume and weight administered with si-SNHG16 were significantly (*P* < 0.05) lower than the si-NC control-treated mice (Figures 7(e) and 7(f)). The results are thus suggestive that the silencing of SNHG16 suppresses the tumor growth in vivo. Additionally, western blot analysis showed that expression of Bax and cleavage of caspase-3 was increased, while the expression of Bcl-2 was decreased upon SNHG16 knockdown (Figure 7(g)) indicative of apoptosis.

## 4. Discussion

At the global level, lung cancer is the leading cause of cancer-related deaths [1–3]. Out of the total lung cancer cases detected per year, more than 85% patients die within the same year [20]. One of the major factors responsible for extremely high mortality of lung cancer is that most of the patients are diagnosed with the malignancy only at stage III or IV [21]. In the present study, the gene expression levels of lncRNA-SNHG16 were found to be correlating with the advancement of lung cancer pathogenesis and thus might be utilized as a vital prognostic tool against the same as has been also proposed for SNHG16 in other human cancers [22, 23]. Further, the distant metastasis is believed to make it exceedingly difficult to combat the malignancy of human lung cancer rendering the current therapeutic measures ineffective [24]. Thus, to devise more efficient and novel anticancer strategies against the lung cancer, it is very crucial to understand the mechanics of this deadly disorder more thoroughly with exploration of various molecular links underlying its growth and progression. The present study showed that lncRNA-SNHG16 regulates the growth of lung cancer cells, and its transcriptional silencing effectively reduced the in vitro cancer cell growth. The tumor-suppressive effect of SNHG16 knockdown was shown to be resulting from the induction of apoptosis in lung cancer cells which further confirms its established role as reported by the previous research investigations [25, 26]. The key finding of the present of the present study was the elucidation of molecular interaction of SNHG16 with human aldehyde dehydrogenase 2 (ALDH2) at posttranscriptional level. The study revealed that the latter is negatively regulated by SNHG16 at posttranscriptional level as proven from luciferase assay and both the gene and protein expression studies of ALDH2. The study made it lucid that repression of ALDH2 at posttranscriptional level by SNHG16 was responsible for exerting the growth regulatory role of latter in lung cancer. The tumor regulatory role of SNHG16 was also evident from the in vivo mice tumorigenesis study. The results suggested that the in vivo tumor growth was reduced through ALDH2 upregulation. The repression of ALDH2 in lung cancer and its tumor promoting role has been worked out previously [27]. It was found that reduced ALDH2 levels impair the pathway of detoxification of acetaldehyde to nontoxic acetic acid and proved how it caused DNA damage and promoted the growth and development of lung cancer [27]. Hence, the present research work is an extension to the established research results about the role of ALDH2 in lung cancer and disclosed the mechanism accounting for its repression in lung cancer to promote the advancement of this deadly disease. Taken together, the results of current study suggest prognostic and therapeutic utility of lncRNA-SNHG16 and its down-stream target ALDH2 against human lung cancer. Nonetheless, it needs to be elucidated whether SNHG16 directly regulates the expression of ALDH2 or via involvement of microRNA. Additionally, findings need to be confirmed by study involving large number of clinical specimen.

## 5. Conclusion

Collectively, the results of current study are supportive of the growth regulatory role of SNHG16 in lung cancer. The study highlights the prognostic and therapeutic role of SNHG16 in lung cancer and advocates the targeting of SNHG16/ALDH2 axis as potential and novel anticancer strategy against the devastating malignancy of human lung cancer.

## Figures and Tables

**Table 1:** Clinicopathological features and expression of SNGH in lung cancer patients.

Characteristics	Lung cancer patients (*n* = 30)	SNGH16 expression		*P* value
		Low (*n* = 13)	High (*n* = 17)	
Age				0.815
60	13	5	8	
60	17	8	9	
Gender				
Male	19	9	10	0.562
Female	11	4	7	
Tumor size				
5 cm	19	7	12	0.311
5 cm	11	6	5	
Lymph node metastasis				
Yes	11	3	8	0.012
No	20	10	10	
Distant metastasis				0.004
Yes	10	4	6	
No	20	9	11	
Clinical stage				0.005
I-11	12	6	6	
III-IV	18	7	11	

**Table 2:** List of primers used in the study.

Primer	Direction	Sequence
SNHG16	Forward	5′-GCAGAATGCCATGGTTTCCC-3′
	Reverse	5′-GGACAGCTGGCAAGAGACTT-3′

ALDH2	Forward	5- GGTGGCTGTAGGAATCTGTCA-3′
	Reverse	5′- AGGGAAAGAGGAAACTCCTGA A-3′

*β*-Actin	Forward	5′-CCTGGATAGC AACGTAC-3′
	Reverse	5′-CACCTTCTACAATGAGCT-3′

GADPH	Forward	5′-TCTCTGCTCCTCCTGTTC-3′
	Reverse	5′-GGTTGAGCACAGGGTACTTTATTGA-3′

## Data Availability

The data regarding this paper is available on request to the author.
